# Genotypic Variation in Resistance Gene-Mediated Calcium Signaling and Hormonal Signaling Involved in Effector-Triggered Immunity or Disease Susceptibility in the *Xanthomonas campestris* pv. *Campestris*–*Brassica napus* Pathosystem

**DOI:** 10.3390/plants9030303

**Published:** 2020-03-01

**Authors:** Md. Al Mamun, Md. Tabibul Islam, Bok-Rye Lee, Van Hien La, Dong-Won Bae, Tae-Hwan Kim

**Affiliations:** 1 Department of Animal Science, Institute of Agricultural Science and Technology, College of Agriculture & Life Sciences, Chonnam National University, Gwangju 61186, Korea; almamun.uoda@gmail.com (M.A.M.); tabib_pha@hotmail.com (M.T.I.); turfphy@hotmail.com (B.-R.L.); hiencnsh87@gmail.com (V.H.L.); 2 Alson H. Smith Jr. Agricultural Research and Extension Center, School of Plant and Environmental Sciences, Virginia Tech, Winchester, VA 22602, USA; 3 Asian Pear Research Institute, Chonnam National University, Gwangju, 61186, Korea; 4 Biomaterial Analytical Laboratory, Central Instruments Facility, Gyeongsang National University, Jinju, 52828, Korea; bdwon@gnu.ac.kr

**Keywords:** jasmonic acid, salicylic acid, *ZAR1*, *TAO1*, *Xanthomonas campestris* pv. *campestris*

## Abstract

To characterize cultivar variation in resistance gene (R-gene)-mediated calcium signaling and hormonal regulation in effector-triggered immunity (ETI) and disease susceptibility, *Xanthomonas campestris* pv. *campestris* (*Xcc*) was inoculated in two *Brassica napus* cultivars (cvs. Capitol and Mosa). At 14 days post inoculation (DPI) with *Xcc*, there was a necrotic lesion in cv. Mosa along with the significant accumulation of H_2_O_2_ and malondialdehyde (MDA), whereas no visual symptom was observed in cv. Capitol. The cultivar variations in the R-gene expressions were found in response to *Xcc*. *ZAR1* is a coiled-coil-nucleotide binding site-leucine-rich repeat (CC-NB-LRR)-type R-gene that is significantly induced in cv. Capitol, whereas toll/interleukin-1 receptor-nucleotide binding site-leucine-rich repeat (TIR-NB-LRR)-type R-gene, *TAO1*, is significantly upregulated in cv. Mosa *Xcc*-inoculated plants. The defense-related gene’s non-race-specific disease resistance 1 (*NDR1*) and mitogen-activated protein kinase 6 (*MAPK6*) were enhanced, whereas calcium-dependent protein kinase (*CDPK5*) and calcium-sensing protein 60g (*CBP60g)* were depressed in cv. Capitol *Xcc* inoculated plants, and opposite results were found in cv. Mosa. The calcium-sensing receptor (*CAS*), calmodulin (*CaM*), expression was induced in both the cultivars. However, the *CAS* induction rate was much higher in cv. Mosa than in cv. Capitol in response to *Xcc*. The phytohormone salicylic acid (SA) and jasmonic acid (JA) levels were significantly higher in cv. Capitol along with the enhanced SA receptors (*NPR3* and *NPR4*) and JA synthesis and signaling-related gene expression (*LOX2, PDF1.2*), whereas the JA level was significantly lower in cv. Mosa *Xcc* inoculated plants. The SA synthesis and signaling-related genes (*ICS1, NPR1*) and SA were present at higher levels in cv. Mosa; additionally, the SA level present was much higher in the susceptible cultivar (cv. Mosa) than in the resistant cultivar (cv. Capitol) in response to *Xcc*. These results indicate that *ZAR1* mediated the coordinated action of SA and JA synthesis and signaling to confirm ETI, whereas *TAO1* enhanced the synthesis of SA through *CAS* and *CBP60g* to antagonize JA synthesis and signaling to cause disease susceptibility in the *Brassica napus*–*Xcc* pathosystem.

## 1. Introduction

Black rot disease of oilseed rape (*Brassica napus*) caused by *Xanthomonas campestris* pv. *campestris* (*Xcc*) is a major threat that reduces the quality and productivity of Brassicaceae crops. Yellow v-shaped necrotic lesions and darkened leaf veins are the most common symptoms [1,2].

Effector-triggered immunity (ETI) is initiated by resistance genes (R-genes) [3,4]. Coiled-coil-nucleotide binding site-leucine-rich repeat (CC-NB-LRR) and toll/interleukin-1 receptor-nucleotide binding site-leucine-rich repeat (TIR-NB-LRR) are two major types of R-genes that regulate the activation of the plant immune response, especially phytohormone signaling to counteract pathogenic infection [5]. Reactive oxygen species (ROS) and calcium signaling play important roles in pattern-triggered immunity (PTI) or ETI [4]. H_2_O_2_ is the primary detectable oxide that is capable of inducing cytosolic Ca^2+^ and calcium-dependent protein kinases (*CDPKs*) during pathogenic attack [4,6,7]. Phytohormone salicyclic acid (SA), a critical messenger, is induced for immune response during plant–pathogen interaction modulated by different Ca^2+^ signaling [8,9,10].

The phytohormones SA and jasmonic acid (JA) are the central regulators of plant immune responses. SA induces the defense mechanism against biotrophic pathogens, whereas JA induces the defense mechanism against necrotrophic pathogens. SA and JA signaling are mutually antagonistic, and the antagonism depends on SA signaling gene nonexpressor of pathogenesis-related gene 1 (*NPR1*) [11]. The fine-tuning of this hormonal signaling is key to activating effective defense responses to hemibiotrophic pathogens such as *Xcc* [7], which has both biotrophic and necrotrophic phases of infection. Liu et al. (2016) [12] reported that the nonexpressor of pathogenesis-related genes 3 and 4 (*NPR3* and *NPR4*) act as SA receptors, which induce JA synthesis and signaling towards ETI against the hemibiotrophic bacterial pathogen *Pseudomonas syringae* pv. *tomato*. The necrotrophic or hemibiotrophic pathogens in the necrotrophic phase of the infection may use SA signaling to induce disease progression by antagonizing JA signaling [13].

We hypothesized that the genotypic difference in the expression of the R-gene might play a significant role in activating the different hormonal signalings through the calcium signaling in resistant and susceptible *B. napus*–*Xcc* interactions. To test this hypothesis, the expressions of two different R-genes *ZAR1* (CC-NB-LRR) and *TAO1* (TIR-NB-LRR) [14] (different calcium signaling genes) were interpreted along with endogenous alteration of JA and SA levels as well as their synthesis and signaling genes in two contrasting *B. napus* cultivars in responses to *Xcc*.

## 2. Results

### 2.1. Development of Symptoms and Oxidative Stress

**Figure 1:**
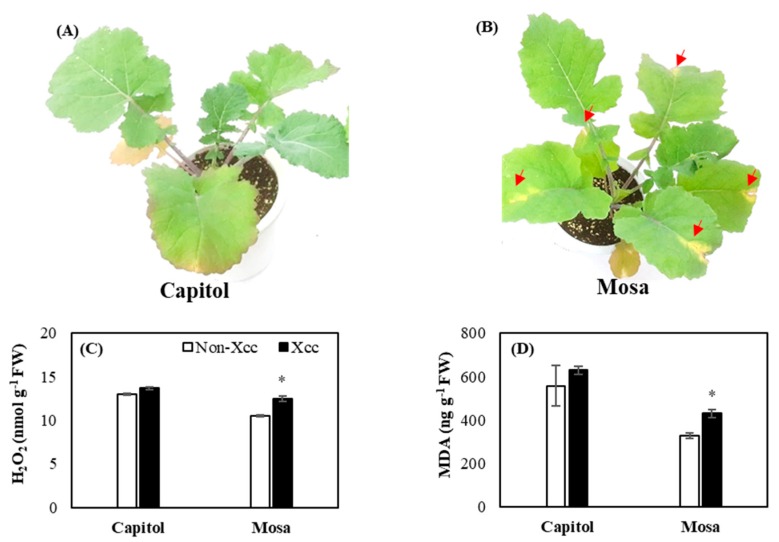
Visible disease symptom development (**A**,**B**), H_2_O_2_ concentration (**C**), and lipid peroxidation level (**D**) in the non-*Xcc*-inoculated (white bar) and *Xcc*-inoculated leaves (black bar) of two *Brassica napus* cultivars. Vertical bars indicate mean ± SE (n=3). Significant difference levels between non-inoculated and *Xcc*-inoculated plants are denoted by * *p* < 0.05, ** *p* < 0.01, *** *p* < 0.001.

In the response to *Xcc*, there were no visual symptoms observed in cv. Capitol (Figure 1A), whereas a visible v-shaped necrotic lesion was clearly observed in cv. Mosa (Figure 1B). In the cultivar Mosa, H_2_O_2_ and lipid peroxidation levels in *Xcc*-inoculated plants were significantly higher (18.8% and 30.8%, respectively) than those of non-*Xcc*-inoculated plants (Figure 1C,D).

### 2.2. Expression of the Resistance Genes and Related Genes

**Figure 2:**
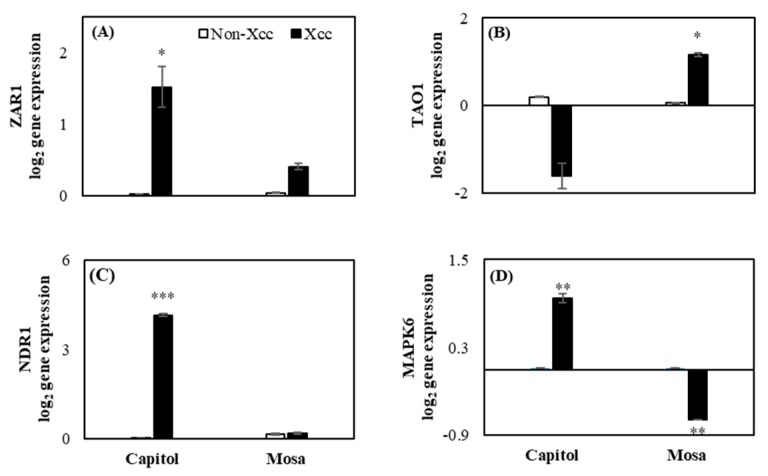
Relative gene expression of Hop (*Hrp*-dependent outer protein) Z-activated resistance 1 (*ZAR1*, **A**) and target of AvrB operation 1 (*TAO1*, **B**), non-race-specific disease resistance 1 (*NDR1,* **C**), mitogen-activated protein kinase 6 (*MAPK6,* **D**) in the non-*Xcc*-inoculated (white bar) and *Xcc*-inoculated leaves (black bar) of two *Brassica napus* cultivars. Vertical bars mean ± SE (n=3). Significant levels (between non-inoculated and *Xcc*-inoculated plants) are denoted by * *p* < 0.05, ** *p* < 0.01, *** *p* < 0.001.

In cv. Capitol, *Xcc* inoculation enhanced the expression of CC-NB-LRR-type R-gene *ZAR1* (Figure 2A). However, in cv. Mosa, TIR-NB-LRR-type R-gene *TAO1* was significantly enhanced by *Xcc* inoculation, whereas it was downregulated in cv. Capitol (Figure 2B). *NDR1* gene (Figure 2C) was significantly induced (102.75-fold) in cv. Capitol, whereas it was not changed in cv. Mosa. *Xcc* inoculation induced *MAPK6* (Figure 2D) significantly (47.5-fold) in cv. Capitol, and it was downregulated in cv. Mosa.

### 2.3. Expression of the Calcium Signaling-Related Genes

**Figure 3:**
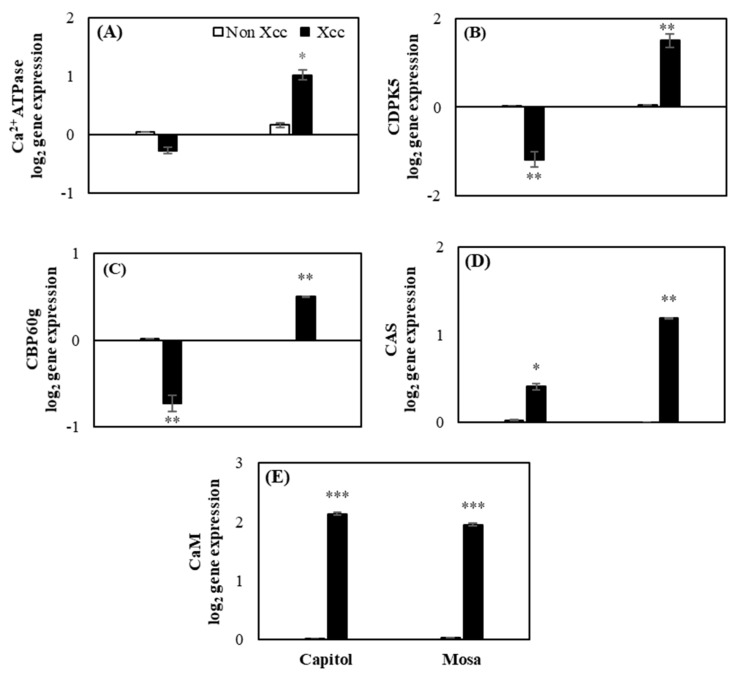
Relative expression of calcium signaling genes. (**A**) *Ca^2+^ATPase*, (**B**) calcium-dependent protein kinase (*CDPK5*), (**C**) calmodulin-binding protein 60g *(CBP60g)*, (**D**) calcium-sensing receptor (*CAS*), (**E**) *calmodulin (CaM)* in the non-*Xcc*-inoculated (white bar) and *Xcc*-inoculated leaves (black bar) of two *Brassica napus* cultivars. Vertical bars mean ± SE (n=3). Significant levels (between non-inoculated and *Xcc*-inoculated plants) are denoted by * *p* < 0.05, ** *p* < 0.01, *** *p* < 0.001.

The calcium sensors *Ca^2+^ATPase*, *CDPK5*, and *CBP60g* were downregulated in cv. Capitol, whereas these were significantly upregulated in cv. Mosa (5.24-fold, 34.71%, 1.00-fold, respectively) by *Xcc* inoculation (Figure 3A,B,C). However, *Xcc* induced the expression of *CAS* and *Calmodulin* (*CaM*) significantly in both cultivars (Figure 3D,E).

### 2.4. Expression of Hormonal Synthesis-Related Genes and Endogenous Content of SA and JA

**Figure 4:**
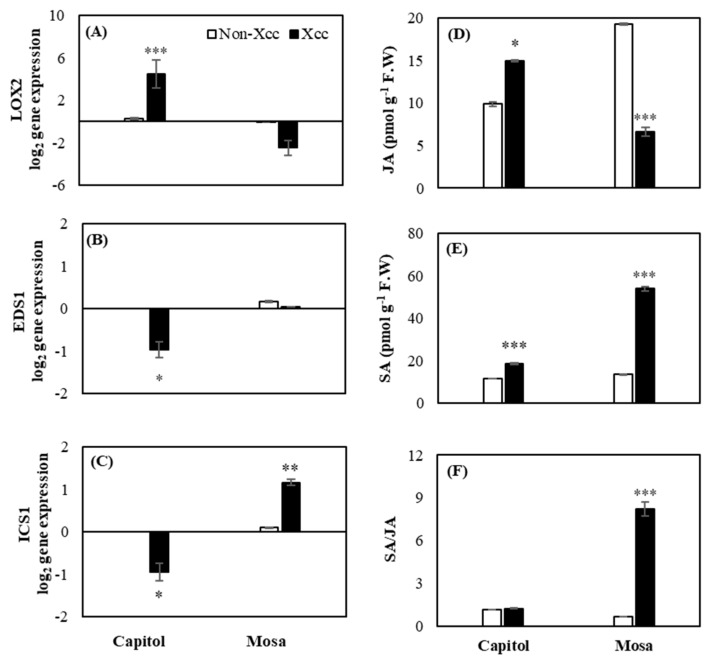
Relative expression of hormonal synthesis-related genes (**A**–**C**) and the endogenous level of jasmonic acid (JA, **D**), salicylic acid (SA, **E**), and SA/JA ratio (**F**) in the non-*Xcc*-inoculated (white bar) and *Xcc*-inoculated leaves (black bar) of two *Brassica napus* cultivars. Vertical bars mean ± SE (n=3). Significant differences between non-inoculated and *Xcc*-inoculated plants are denoted by * *p* < 0.05, ** *p* < 0.01, *** *p* < 0.001.

JA synthesis-related gene lipoxygenase 2 (*LOX2*) was significantly (*p* < 0.001) enhanced by *Xcc* inoculation in cv. Capitol, whereas it was depressed (-40.16%) in cv. Mosa when compared to the respective non-*Xcc*-inoculated plants (Figure 4A). *Xcc* inoculation significantly (*p* < 0.05) depressed two SA synthesis-related genes, *EDS1* and *ICS1,* in cv. Capitol. However, a significant enhancement of *ICS1* expression was observed only in cv. Mosa (Figure 4B,C).

*Xcc* inoculation increased (*p* < 0.05) the endogenous level of JA in cv. Capitol, while it significantly decreased (*p* < 0.001) in cv. Mosa (Figure 4D). *Xcc* inoculation significantly increased the SA content in both cultivars. The increase of SA was much higher in cv. Mosa (2.9-fold) than in cv. Capitol (1.2-fold) (Figure 4E). *Xcc* inoculation-induced alteration in the endogenous SA and JA levels was much more distinct in cv. Mosa (5.3-fold of SA/JA ratio), whereas the ratio was not significantly changed in cv. Capitol (Figure 4F).

### 2.5. Expression of Hormonal Signaling-Related Genes

**Figure 5:**
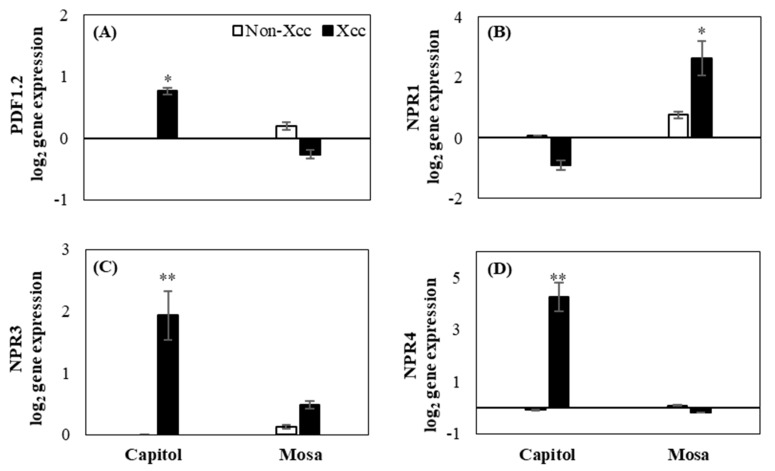
Relative expression of hormonal signaling genes. (**A**) Plant defensing factor 1.2 (*PDF1.2*), (**B**) non-expresser of pathogenesis-related (PR) genes 1 (*NPR1*), (**C**) *NPR3*, and (**D**) *NPR4* in the non-*Xcc*-inoculated (white bar) and *Xcc*-inoculated leaves (black bar) of two *Brassica napus* cultivars. Vertical bars mean ± SE (n=3). Significant levels (between non-inoculated and *Xcc*-inoculated plants) are denoted by * *p* < 0.05, ** *p* < 0.01, *** *p* < 0.001.

In cv. Capitol, *Xcc* inoculation highly enhanced the expression of JA signaling-related gene plant defensin 1.2 (*PDF1.2*) (Figure 5A), whereas enhanced expression of the SA signaling gene *NPR1* was observed in cv. Mosa (Figure 5B). SA receptor genes (*NPR3* and *NPR4*) were significantly expressed in cv. Capitol (Figure 5C,D).

## 3. Discussion

During plant–pathogen interactions, H_2_O_2_ is the primary detectable oxide that can initiate the destruction of the challenged plant cell through lipid peroxidation or programmed cell death [6,7]. In the present study, cultivar variation in symptoms and oxidative stress development was observed (Figure 1A,B), as shown by a yellow v-shaped necrotic lesion along with enhanced H_2_O_2_ (Figure 1C) and lipid peroxidation (Figure 1D) in cv. Mosa, whereas no visible symptoms were observed in cv. Capitol in response to *Xcc* inoculation. ROS (especially H_2_O_2_ production) relies on calcium signaling in response to pathogens. The primary detectable oxide H_2_O_2_ is capable of inducing cytosolic Ca^2+^ during pathogenic attack. Interestingly, the full activation of respiratory burst oxidase homolog protein D (RBOHD) for ROS production requires phosphorylation by Ca^2+^-induced calcium-dependent protein kinases (CDPKs) [4,6,7], as we found that different calcium signaling genes were upregulated in cv. Mosa *Xcc*-inoculated plants along with H_2_O_2_ and MDA (Figure 1C,D and Figure 3).

In our previous study, it was found that cultivar variation in hormonal status regulates the development of *Xcc* symptoms in different *B. napus* cultivars [7,13,15]. In addition, it has been reported that the plant immune system might play a significant role in fine-tuning hormonal signaling to activate effective immune responses against pathogen infection [11,16].

Plants possess a multilayered immune system to protect them from pathogenic infection [17,18,19]. Pattern recognition receptors (PRRs) initiate the recognition of pathogen-associated molecular patterns (PAMPs) to induce pattern-triggered immunity [17]. ETI is a long-lasting defense response and has been known to be activated by the interactions between the pathogen effector and plant R-genes. During the plant–pathogen interaction, different calcium signaling genes are induced towards plant defense [4,10,20]. In the present study, we hypothesized that the R-genes are involved in regulating hormonal signaling through calcium signaling in the resistant or susceptible interaction in the *B. napus*–*Xcc* pathosystem. We thus analyzed the expressions of two types of R-genes, *ZAR1* and *TAO1*, in two *B. napus* cultivars, contrasting disease susceptibility against *Xcc* infection. The R-genes are usually nucleotide-binding/leucine-rich-repeat (NLR) receptors [21], and two sub-families of these R-genes could be distinguished as CC-NB-LRR and TIR-NB-LRR, which regulate plant defense through calcium and phytohormonal signaling [5].

*ZAR1* is one of the CC-NB-LRR R-genes that regulates ETI in response to *Xcc* through the binding complex to recognize the bacterial effector in *Arabidopsis* [22]. It has also been reported that CC-NB-LRR-type R-genes regulate the coordinated actions of SA and JA responses to induce ETI against hemibiotrophic pathogens [12]. For ETI induction, *NDR1* and MAPKs are important genes that are activated by the induction of R-genes in biotic stress, and in CC-NB-LRR type R-genes, MAPKs are responsible for regulating SA signaling for inducing ETI [5,23]. In this study, we found that *NDR1* and *MAPK6* (Figure 2C,D) were significantly upregulated along with *ZAR1* (Figure 2A). This indicates that *ZAR1* might upregulate *NDR1* and *MAPK6* after *Xcc* inoculation in cv. Capitol. However, *TAO1* belongs to another group of R-genes (TIR-NB-LRR) responsible for disease resistance by inducing SA signaling [14,15]. *Ca^2+^ATPase* is an important sensor in calcium fluctuation in several types of stresses [24], and in this study, it was regulated in cv. Mosa with *Xcc* inoculation (Figure 3A). Another calcium signaling gene, *CDPK5*, is an important calcium sensor [6,7,25] in biotic stress that is also induced in cv. Mosa (Figure 3B) after *Xcc* inoculation. This indicates that *TAO1* (Figure 2B), after *Xcc* inoculation, induced the expression of *Ca^2+^ATPase* and *CDPK5* (Figure 3A,B) along with symptom development in cv. Mosa. Calcium-sensing receptors and signaling genes are involved in phytohormonal synthesis for the plant immunity system [8,9]. Positive interactions between *CaM* and *CBP60g* regulate SA synthesis and signaling through the *ICS1* in disease resistance [8,9]. Another report indicated that unknown calcium signaling-induced *CAS* is involved in SA biosynthesis through *ICS1* for mediating ETI of PTI [9,26]. In the current study, *CAS* and *CaM* (Figure 3D,E) gene expressions were significantly induced in both cultivars along with SA (Figure 4E), whereas *CBP60g* (Figure 3C) was highly expressed in cv. Mosa. The results indicate that calcium signaling genes *Ca^2+^ATPase*, *CDPK5*, and *CaM* (Figure 3A,B,E) might be induced by the induction of *TAO1* with interaction with H_2_O_2_ in cv. Mosa (Figure 1C and Figure 2B). These calcium signaling genes might have induced *CBP60g* and *CAS* for SA synthesis and signaling after *Xcc* inoculation in cv. Mosa (Figure 3C,D and Figure 4E), whereas *ZAR1* (Figure 2A) might induce the *CAS* and *CaM* (Figure 3D,E) that mediated SA synthesis and signaling in cv. Capitol. However, the question of how *CAS* and *CaM* mediate SA synthesis and signaling still needs to be answered.

Phytohormones SA and JA are the usual central regulators of the plant defense system against biotrophic and necrotrophic pathogens, respectively [7,16,27]. In the present study, the expression of R-genes in response to *Xcc* infection was associated with the endogenous hormonal status in the resistant or susceptible *B. napus*–*Xcc* interaction, as shown by a distinct accumulation of JA in cv. Capitol (Figure 4D), while SA in cv. Mosa (Figure 4E) resulted in a much higher SA/JA ratio in cv. Mosa (Figure 4F). Previously, we found with six different *B. napus* that a higher SA/JA ratio was symptomatic of *Xcc* disease [7] and that the JA-mediated phenolic metabolite accumulation, especially in the cell wall-bound form, was an important feature of disease resistance [16]. The present data confirmed that JA induction with an antagonistic depression of SA (thus maintaining proper SA/JA ratio) would be part of the resistance mechanism against the hemibiotrophic pathogen *Xcc* in *B. napus.*

Furthermore, in the resistant cultivar Capitol, the enhanced expression of *ZAR1* (Figure 2A) was concomitant with the enhanced expression of JA synthesis-related *LOX2* (Figure 4A) and JA signaling *PDF1.2* (Figure 5A) genes, accompanied by higher accumulations of JA (Figure 4D). Interestingly, enhanced expression of *ZAR1* in cv. Capitol was accompanied by enhanced expression of SA receptor genes *NPR3* and *NPR4* (Figure 5C,D). It has been reported that the *RPS2*, a CC-NB-LRR-type R-gene, induced *NPR3* and *NPR4* and activated JA synthesis via the degradation of *JAZ1* proteins in response to hemibiotrophic pathogen infection [13]. In addition, higher accumulation of SA depressed the expression of *NPR3* and *NPR4* [28]. In the susceptible cultivar cv. Mosa, the enhanced expression of TIR-NB-LRR-type R-gene *TAO1* (Figure 2B) coincided with the enhanced expression of SA synthesis-related genes *ICS1* (Figure 4C) along with *CBP60g* and *CAS* (Figure 3C,D). Higher disease susceptibility in cv. Mosa (Figure 1B) was characterized by the enhanced expression of *TAO1*-mediated SA-dependent gene *NPR1* (Figure 5B), which was accompanied by a higher SA/JA ratio (Figure 4F), in accordance with previous reports [5,14,29].

**Figure 6:**
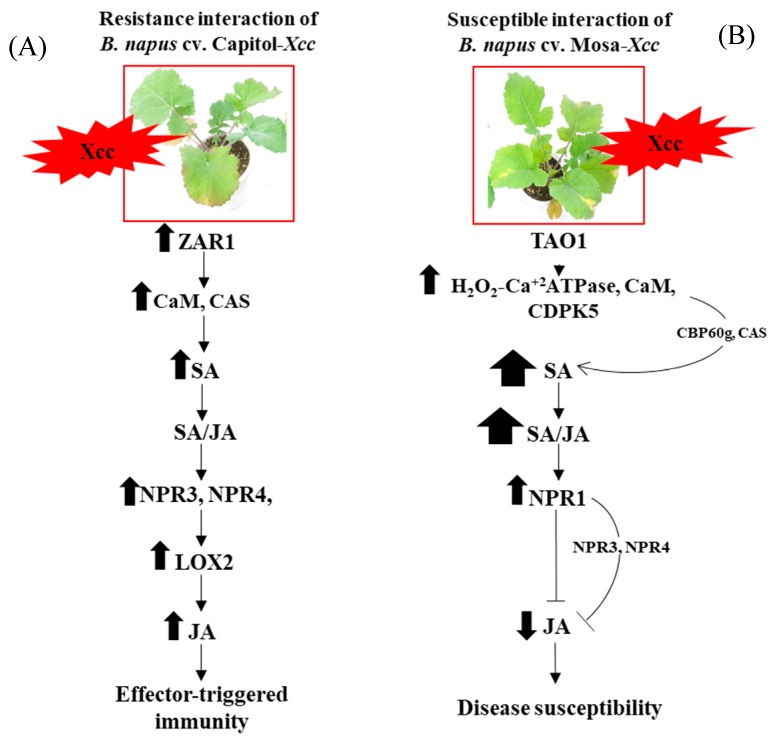
Outline of R-gene-mediated hormonal signaling in effector-triggered immunity (ETI) in *Brassica napus* cv. Capitol (**A**) and disease susceptibility in *Brassica napus* cv. Mosa (**B**) in response to *Xcc* inoculation. The impact of treatment on the measurement was expressed by the thickness of each arrow.

This report, as far as we know, is the first to provide evidence that CC-NB-LRR R-gene *ZAR1* is involved in mediating *CAS* and *CaM* to initiate SA synthesis and signaling-regulated disease-resistant interaction by JA synthesis and signaling (Figure 6A), whereas the TIR-NB-LRR-type R-gene *TAO1*-mediated SA accumulation by *CAS* and *CBP60g* with an antagonistic depression of JA and is part of disease susceptibility (Figure 6B) in the *B. napus*–*Xcc* pathosystem.

## 4. Materials and Methods

### 4.1. Plant Material and Bacterial Inoculation

The seedlings of two *B. napus* cultivars (cvs. Capitol and Mosa) were grown in plastic pots. After six leaf stages, the plants were divided into two groups of each cultivar. One group was the control group, and another group was the experimental group with pathogen inoculation. The pathogenic bacterial (*Xanthomonas campestris* pv. *campestris*) strain was collected from the Korean Agricultural Culture Collection and was cultured in Yeast dextrose calcium carbonate (YDC) in an agar plate at 30 °C for 48 h. The bacterial concentration was then adjusted with 10^8^ CFU/mL, and the plants’ leaves were inoculated with the bacteria by clipping the leaf edges near the veins using mouth–tooth forceps. The experiment was conducted with a completely randomized design with three biological replications. After 14 days of inoculation, six leaves of each plant were sampled from both pathogen-inoculated or control (non-inoculated) plants in the same leaf rank order. Six combined leaves were considered as a biological replicate of each treatment. The collected samples were immediately frozen in liquid nitrogen and stored in a deep freezer (−80 °C) for further analysis.

### 4.2. Determination of Hydrogen Peroxide (H_2_O_2_) and Lipid Peroxidation

Hydrogen peroxide levels were measured according to the method described by Venisse et al., 2019 [30]. The concentration of malondialdehyde (MDA) was measured to determine the lipid peroxidation level, using the method described by Lee et al., 2007 [31].

### 4.3. Phytohormone Analysis

Quantitative analysis of jasmonic acid and salicylic acid in the leaf tissue was performed according to Pan et al., 2010 [32]. JA and SA extracts from 50 mg of well-ground leaves were injected into a reverse-phase C18 Gemini high-performance liquid chromatography (HPLC) column for HPLC-electrospray ionization tandem mass spectrometry (HPLC-ESI-MS/MS) analysis. Agilent 1100 HPLC (Agilent Technologies), Waters C18 column (150 × 2.1 mm, 5 μm), and API3000 MSMRM (Applied Biosystems) were used for the analysis.

### 4.4. Isolation of Total RNA and Quantitative Real-Time PCR

Total RNA was isolated from 200 mg of leaf tissue using an RNAiso Plus (TAKARA BIO INC., Nojihigashi 7-4-38, Kusatsu, Shiga 525-0058, Japan). The GoScript Reverse Transcription System (TAKARA BIO INC., Nojihigashi 7-4-38, Kusatsu, Shiga 525-0058, Japan) was used to synthesize complementary DNA (cDNA) from the RNA. A light cycle real-time PCR detection system was used to quantify the gene expression level. The PCR reactions were initiated at 95 °C for 5 min, and then, 45 cycles of 95 °C for 30 s, 54–59 °C for 30 s (depending on target primers), and 72 °C for 30 s were initiated, with the final extension at 72 °C for 5 min. The gene-specific primers used for the qRT-PCR are given in Table S1. The qRT-PCR reactions were performed in duplicate for each of the three independent samples.

### 4.5. Statistical Analysis

A completely randomized design was used with three replicates for each treatment. Duncan’s multiple range test was performed to compare the means of separate replicates. Statistical significance was determined at *p* < 0.05. SAS 9.1.3 (SAS Institute Inc., Cary, NC, USA) was used to perform the statistical analysis of all the measurements.

## Supplementary Materials

The following are available online at https://www.mdpi.com/2223-7747/9/3/303/s1, Table S1: Specific primers used for qRT-PCR.
