# Supplementary material for: Genotypic Variation in Resistance Gene-Mediated Calcium Signaling and Hormonal Signaling Involved in Effector-Triggered Immunity or Disease Susceptibility in the Xanthomonas campestris pv. Campestris–Brassica napus Pathosystem
Source: Plants (Basel). 2020 Mar 1;9(3):303. doi: 10.3390/plants9030303 (PMC7154883; doi:10.3390/plants9030303)
Supplement: Supplementary file 1 [file plants-09-00303-s001.pdf]

plants

MDPI

Supplementary Table S1. Specific primers used for qRT-PCR.

Target gene.	GenBank Number	Forward sequence	Reverse sequence
BnZAR1	XM_013789409.2	5´-GAGGAGAAGAAGCCGTTGTG-3´	5´-GCCAGCCATTGGTAATCTGT-3´
BnTAO1	XM_013826937.2	5´-GGAATCCCCACATGTTTTTG-3´	5´-GTCTGGAAGCTGTGGGAGAG-3´
BnEDS1	EU694108.1	5´-CAGGACATTCCACAGGAGGT-3´	5´-AACCGGCTCCAGTTCTCTCT-3´
BnLOX2	XM_013846955.1	5´-GTGGGTGCCATCAGAGTTTT-3´	5´ GTCTCCAGCTCCTGTTTTCG-3´
BnICS1	XM013887885	5´-TCAATCCCAGAACGAGATCC -3´	5´- GACAGAAACCTTCGGATGGA -3´
BnNPR1	EF613226.1	5´-TGAGAACATTGCCAAGCAAG-3´	5´-CAACAGCAAAATGGAGAGCA-3´
BnNPR3	XM_013826225.2	5´-CCAAAGTTGTTGCCGAGATT-3´	5´-TTCGCAGCTGTTTTCGTATG-3´
BnNPR4	XM_013878638.2	5´-CACAGACGAGGTGGCTGATA-3´	5´-GCCACAACTTTGGGATCACT-3´
BnPDF 1.2	AY884023.1	5´-TGTTTTTGCTGCTTTTGGTG-3´	5´-TCGAATGCACTGATTCTTGC-3´
BjCa^{+2}ATPase	EU143656.1	5´- CGAGTTTAACGCGAGGAGTC -3´	5´- AATGCAAACTCCCCACTGTC -3´
BnCDPK5	NM_001315883.1	5´-GCAGCAATGTCTTCTGGTGA-3´	5´-GCAGCAATGTCTTCTGGTGA-3´
BnCAS	XM_013821844.2	5´-GGCGTGGAAAATATTGCTGT-3´	5´-GCCATGCAAGCTCCATTTAT-3´
BnCBP60g	XM_013805373.2	5´-AGCTCACTTCACGGAGGAAA-3´	5´-TCACCACCGGTTAACCTAGC-3´
BnCaM	XM_013826798.2	5´-GGGAACGGGACCATAGATTT-3´	5´-TCTCCCCAAGGTTTGTCATC-3´
BnMAPK6	XM_013884849.2	5´-GCTAGCTCCATGGGACAGAG-3´	5´-GAGCAGTTGGTGGTGGATTT-3´
BnNDR1	XR_002655787.1	5´-CCCTCTTTCTCCTCCAATCC-3´	5´-TTCTCCTTGAACGCAGAGGT-3´
ACTIN	AF111812	5´-GATTCCGTTGCCCTGAAGTA-3´	5´-GCGACCACCTTGATCTTCAT-3´

All primers were designed directly from sequences in the public database.
